# Quantitative Analysis of 137 MRI Images in Hydrocephalic Dogs

**DOI:** 10.3390/vetsci12030221

**Published:** 2025-03-02

**Authors:** Hao Zhuang, Qiqing Yang, Lin Zhang, Xiaosong Xiang, Dandan Geng, Qiyun Xie, Changmin Hu

**Affiliations:** 1 Department of Clinical Veterinary Medicine, College of Veterinary Medicine, Huazhong Agricultural University, Wuhan 430070, China; zhuanghao2019@163.com (H.Z.); xieqy0326@126.com (Q.X.); 2 Shanghai AiLv Animal Hospital, Shanghai 200235, China; 13901782616@163.com; 3 Shanghai ChongQin Animal Hospital, Shanghai 200235, China; shcqpet@163.com; 4 Shanghai Miero Pet Hospital, Shanghai 200235, China; xiaosongxiang@sina.com (X.X.); dandangeng1124@sina.com (D.G.); 5 The Veterinary Teaching Hospital, College of Veterinary Medicine, Huazhong Agricultural University, Wuhan 430070, China

**Keywords:** quantitative analysis, magnetic resonance imaging, hydrocephalus, lateral ventricles

## Abstract

With the increasing popularity of dogs as pets, cases of hydrocephalus have risen significantly. Due to the ongoing challenges in the diagnosis and treatment of hydrocephalus, advancements in magnetic resonance imaging (MRI) technology have greatly enhanced the diagnostic capabilities in small animal clinical practice. Assessing ventricular size is crucial for the clinical management of hydrocephalus and other neurological disorders. However, methods for quantifying ventricular size and evaluating the severity of hydrocephalus requires further optimization. This study involved 137 hydrocephalus and 17 normal dogs. In hydrocephalus cases, the maximum percentage of the ventricle height to brain height (H-max%) was correlated with the area (A-max%) and volume (V-max%). Equations were calculated based on these findings, showing that the percentage of height can effectively represent the percentage of area and volume, which can indicate the diagnosis and monitoring of hydrocephalus prognosis.

## Simple Summary

The purpose of this study was to evaluate the lateral ventricles of hydrocephalic patients using quantitative analysis methods. We measured the height, area, and volume of the lateral ventricles and brain in 154 dogs (17 normal and 137 hydrocephalus cases) using magnetic resonance imaging (MRI), and calculated the percentage of the ventricle measurements relative to the brain. Since there were no significant differences in area and volume between normal animals and those with hydrocephalus, while a significant difference in height was observed, we chose the maximum percentage of the ventricle height to brain height (H-max%) as the core reference indicator. In hydrocephalus cases, H-max% was correlated with the area (A-max%) and volume (V-max%).

## 1. Introduction

The animal brain consists of several major structures, including the cerebrum, brainstem, cerebellum, and ventricular system [1]. The ventricular system includes the left and right lateral ventricles, the third ventricle, and the fourth ventricle. In dogs, cerebrospinal fluid is produced by these four ventricles at a rate of 0.047 mL/min [2]. Hydrocephalus results from a dynamic imbalance between the production and absorption of cerebrospinal fluid, enlarging the ventricles and the subarachnoid space [3]. In 1919, Walter E. Dandy developed a preliminary classification of hydrocephalus using the dye method. Today, hydrocephalus can be categorized into congenital or acquired, communicating or obstructive, and intra-ventricular or extra-ventricular types, as referenced in humans [4].

The primary clinical symptoms of hydrocephalus include abnormal head enlargement, thinning of the skull, expansion of the fontanelles, and separation of the sutures, resulting from increased intracranial pressure. These symptoms may manifest as ataxia, vision deficits, obtundation, pain, strabismus, skull conformation, behavioral changes, disorientation, head tilt, nystagmus, and others [5]. Diagnosing hydrocephalus typically requires imaging examinations for confirmation, such as magnetic resonance imaging (MRI) [6,7,8]. Additionally, MRI allows for detailed studies of blood flow and cerebrospinal fluid dynamics [9,10].

Generally, a height ratio of 15% is used as a diagnostic criterion for hydrocephalus [11]. In 2001, Esteve-Ratsch studied the percentage of the ventricular area in the cerebral hemisphere of Yorkshire Terriers, classifying the asymmetry of the left versus right ventricles into three categories [12]. In 2010, Woo et al. proposed the ventricular-to-brain volume (VV/BV) percentage. Normal dogs exhibited a ventricular brain ratio (VBHR) of less than 25%, a ventricular brain area ratio (VBAR) of less than 7%, and a ventricular brain volume ratio (VBVR) of less than 5%. In contrast, dogs with hydrocephalus had a VBHR greater than 20%, a VBAR greater than 7%, and a VBVR greater than 5% [13]. In 2015, Steffi Laubner showed that a VBA index (the ratio of the ventricular width to the brain width in the coronal image) of more than 0.6, accompanied by elevation of the corpus callosum, deformation of the thalamic adhesion, periventricular edema, expansion of the olfactory recess, thinning of the sulci and subarachnoid space, or rupture of the internal capsule near the tail nucleus, is highly suggestive of clinically significant lateral ventricular expansion caused by increased intracranial pressure [14]. In humans, the Evans index is primarily used, which refers to the ratio of the widest diameter of the frontal horn of the lateral ventricle to the widest diameter of the skull in the same plane on a coronal image [15].

Previous studies have primarily focused on a limited number of samples (10–20 cases) and concentrated on specific breeds, such as German Shepherds or Yorkshire Terriers [12,13,14]. In this study, we conducted a retrospective analysis of 137 cases of hydrocephalus. We performed a quantitative analysis of the height, area, and volume percentage at the thalamic adhesion level using MRI images, without differentiating by the breed, sex, or age of the animals. Our findings demonstrate a strong correlation and provide new insights into the diagnosis of this condition.

## 2. Materials and Methods

### 2.1. Patient Selection and Data Acquisition

MRI images of dogs diagnosed with hydrocephalus were retrospectively analyzed from four referral central animal hospitals in Shanghai and Wuhan, China. The diagnosis was confirmed by a neurologist and imaging specialist certified by the Chinese Veterinary Medical Association. The inclusion criteria required a definitive diagnosis of hydrocephalus based on clinical symptoms (e.g., typical neurological signs such as circling or forebrain dysfunction), neurological examination, and MRI images (TR 615 ms; TE 9.5 ms; slice thickness, 3 mm/5 mm). Ventricular enlargement was assessed according to the criteria described by Laubner et al. (2015), which noted a greater proportion of intracranial volume occupied by the lateral ventricles in cases of ventriculomegaly. In total, 17 normal and 137 hydrocephalus brain MRI images, along with clinical symptoms and basic information, were collected. The study was approved by the Huazhong Agricultural University Animal Ethical and Welfare Committee, approval number HZAUDO-2024-0028. All animal owners signed the informed consent forms [14].

### 2.2. MRI Image Height Processing of Normal and Hydrocephalus Cases

**Figure 1:**
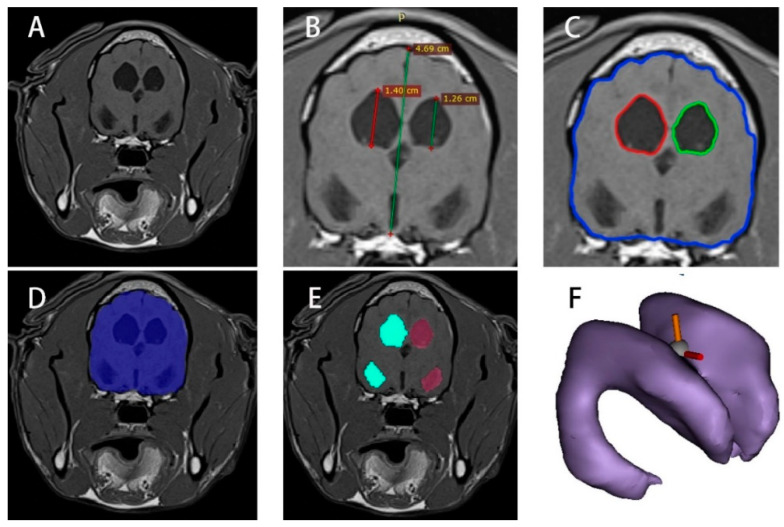
MRI image analysis procedure. (**A**): original MRI image; (**B**): measured heights at the thalamic adhesion level; (**C**): drawing of the ventricle and brain areas; (**D**): ROI extraction of the brain; (**E**): ROI extraction of left and right ventricles; (**F**): 3D model of the lateral ventricle.

T1 images in the DICOM (Digital Imaging and Communications in Medicine) format were imported into RadiAnt Viewer 2020.2 (64-bit) to identify the transverse images at the thalamic adhesion level (Figure 1B). The images were used to measure the height of the brain, denoted as BH (brain height); the height of the left ventricle, recorded as VHl (left ventricular height); and the height of the right ventricle, recorded as VHr (right ventricular height).

### 2.3. MRI Image Area Processing of Normal and Hydrocephalus Cases

The DICOM-format T1 images were imported into ImageJ 1.52a to identify transverse images at the thalamic adhesion level (Figure 1C). The “Wand Tool” function was utilized, with the tolerance set to approximately 10 (pixel threshold algorithm). The region of interest (ROI) for the ventricles and the brain was outlined. The “Brush Selections” function was employed for detailed processing of the ROI, achieving a relatively accurate delineation of the brain area (BA) and ventricle areas (VAl, VAr). The corresponding data were calculated using the “Analyze” and “Measure” functions.

### 2.4. MRI Image Volume Processing of Normal and Hydrocephalus Cases

The DICOM files were imported into Mimics (Materialise Co., Ltd., Leuven, Belgium). A new mask was created using the “Threshold” to select the ROI. The ROI range was refined by editing the mask, and modification tools were employed to accurately outline the ROI, as illustrated in Figure 1D,E. The “Boolean Operations”, “Region Growing”, “Smart Fill”, and “Intelligent Expansion” functions were used next for image modification. A 3D model was then generated and saved as an “xxx.sty” file (Figure 1F). This model was utilized to create segments and perform volume measurements for the entire brain (BV), left lateral ventricle (VVl), and right lateral ventricle (VVr).

### 2.5. Quantitative Analysis of Normal and Hydrocephalus Cases

The one-dimensional height, two-dimensional area, and three-dimensional volume of the left and right ventricles were quantitatively analyzed using MRI image segmentation. The percentages of the left and right ventricles to the brain were calculated and recorded as VHl%, VHr%, VAl%, VAr%, VVl%, and VVr% for both normal and hydrocephalus cases. Additionally, the maximum percentages of height (H-max%), area (A-max%), and volume (V-max%) were determined for the hydrocephalus group.

### 2.6. Regression Analysis of Hydrocephalus Cases

For statistical analysis, the hydrocephalus group and the normal group were first compared using the Mann–Whitney U test for non-parametric data analysis, as the data did not meet the assumptions of normal distribution. Correlation analysis was performed using Spearman’s rank correlation coefficient, for example, to assess the relationships between the maximum percentages of ventricular height, area, and volume. Regression equations were then formulated based on the strongest correlations identified. All analyses were conducted using SPSS version 26.0 and GraphPad Prism version 8.0, with *p* < 0.05 representing a significant difference.

## 3. Results

### 3.1. Retrospective Analysis of Normal and Hydrocephalus Cases

There were no significant differences in age and body weight between the normal group and the hydrocephalus group (*p* > 0.05). Similarly, there were no significant differences in the proportions of gender and the breed group. The top three clinical symptoms in the hydrocephalus group were as follows: disorientation (27.74%), ataxia (14.60%), and head tilt (9.49%) (Table 1).

### 3.2. Left and Right Ventricle and Brain Heights in Normal and Hydrocephalus Cases

In a series of hydrocephalic cases, the distribution characteristics of the heights of the left and right lateral ventricles at the thalamic adhesion level were as follows: the height of the left lateral ventricle (VHl) was 8.99 ± 5.42 mm, while the height of the right lateral ventricle (VHr) was 8.59 ± 5.08 mm. The height of the brain (BH) was 31.81 ± 12.48 mm. The data for the normal group are shown in Table 2.

### 3.3. Left and Right Ventricles and Brain Areas in Normal and Hydrocephalus Cases

In the hydrocephalus group, the distribution characteristics of the left and right lateral ventricle areas and the brain area at the thalamic adhesion level were as follows: the area of the left lateral ventricle (VAl) was 123.10 ± 215.00 mm^2^; the area of the right lateral ventricle (VAr) was 119.80 ± 211.70 mm^2^; and the area of the brain (VA) was 1747.00 ± 985.30 mm^2^. The data for the normal group are presented in Table 3.

### 3.4. Left and Right Ventricle and Brain Volumes in Normal and Hydrocephalus Cases

In the hydrocephalus group, the distribution characteristics of the volumes of the left and right lateral ventricles and the brain were as follows: the volume of the left lateral ventricle (VVl) was 2755.00 ± 6247.00 mm^3^; the volume of the right lateral ventricle (VVr) was 2646.00 ± 6194.00 mm^3^; and the volume of the brain (VV) was 50,906.00 ± 31,346.00 mm^3^. The data for the normal group are presented in Table 4.

### 3.5. Quantitative Analysis Results for Normal and Hydrocephalus Cases

**Figure 2:**
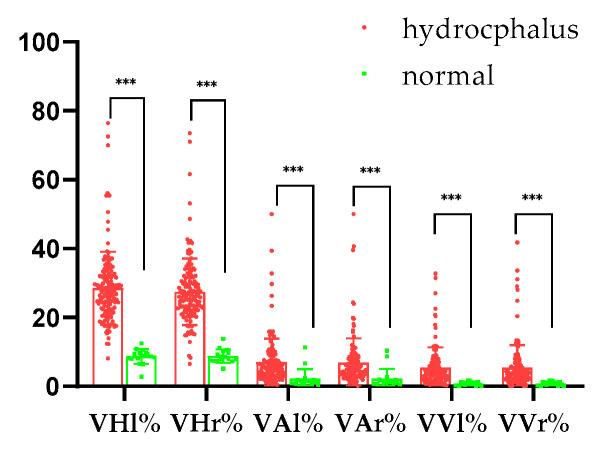
Percentages of height, area, and volume. VHl%: the percentage of left ventricle height to brain height; VHr%: the percentage of right ventricle height to brain height; VAl%: the percentage of left ventricle area to brain area; VAr%: the percentage of right ventricle area to brain area. VVl%: the percentage of left ventricle volume to brain volume; VVr%: the percentage of right ventricle volume to brain volume. ***: *p* < 0.001.

In animals with hydrocephalus, the percentage of left ventricle height to brain height (VHl%) was 28.56 ± 10.58%, while the percentage of right ventricle height to brain height (VHr%) was 27.46 ± 9.67%. The percentage of left ventricle area to brain area (VAl%) was 6.93 ± 6.94%, and the percentage of right ventricle area to brain area (VAr%) was 6.91 ± 7.05%. For volume, the percentage of left ventricle volume to brain volume (VVl%) was 5.36 ± 5.96%, and the percentage of right ventricle volume to brain volume (VVr%) was 5.38 ± 6.60%. There were significant differences between normal and hydrocephalus cases (*p* < 0.001; Figure 2).

**Figure 3:**
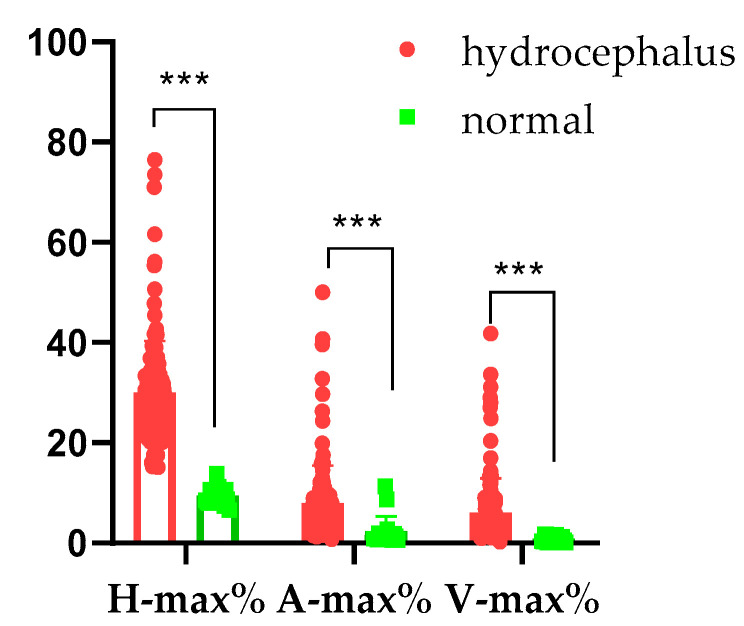
Maximum percentages of hydrocephalus and normal brains. H-max%: the maximum percentage of the ventricle height to brain height; A-max%: the maximum percentage of ventricle area to brain area; V-max%: the maximum percentage of the ventricle volume to brain volume. ***: *p* < 0.001.

In animals with hydrocephalus, the maximum percentage of the ventricle height to brain height (H-max%) was 28.56 ± 10.58% (range: 15.14~76.44%), the maximum percentage of the ventricle area to brain area (A-max%) was 7.94 ± 7.54% (range: 0.76~50.00%), and the maximum percentage of the ventricle volume to brain volume (V-max%) was 6.10 ± 6.80% (range: 0.30~41.75%). Conversely, the normal animals’ H-max% was 9.44 ± 1.70% (range: 6.51–13.78%), their A-max% was 2.36 ± 2.96% (range: 0.60–11.30%), and their V-max% was 0.86 ± 0.58% (range: 0.12–1.62%). There were significant differences between the normal and hydrocephalus cases (*p* < 0.001; Figure 3).

### 3.6. Regression Analysis Result for the Hydrocephalus Cases

The data analysis of hydrocephalic animals showed that the correlation coefficient between the maximum percentage of height and area was R = 0.894, while the correlation coefficient between the maximum percentage of height and volume was R = 0.792. Compared with other correlation coefficients, these values represent an optimal combination (Table 5).

**Figure 4:**
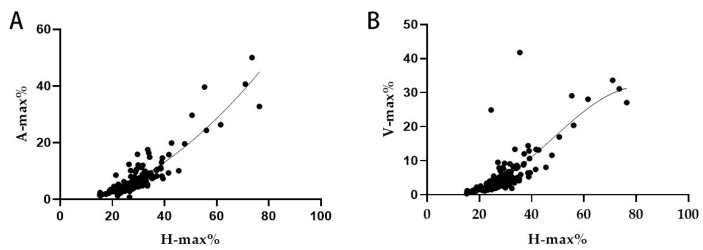
Regression plots of height versus area and height versus volume. H-max%: the maximum percentage of height; A-max%: the maximum percentage of area; V-max%: the maximum percentage of volume. (**A**): Height and area regression plot; (**B**): Height and volume regression plots.

A linear regression model was established (Figure 4) describing the relationship between the maximum percentage of the ventricle height to brain height (X = H-max%) and the maximum percentage of the ventricle area to brain area (Y = A-max%). The established equation was as follows: Y = −2.723 + 0.1451X + 0.006262X^2^. Additionally, the regression equation for the relationship between the maximum percentage of the ventricle height to brain height (X = H-max%) and the maximum percentage of the ventricle volume to brain volume (Y = V-max%) was Y = 7.027 − 0.7862X + 0.03074X^2^ − 0.0002134X^3^.

## 4. Discussion

Hydrocephalus presents with a range of clinical symptoms, primarily characterized by neurological signs and behavioral abnormalities [16]. In the hydrocephalus group in this study, the three most common clinical symptoms were disorientation (27.74%), ataxia (14.60%), and head tilt (9.49%). The balance between cerebrospinal fluid (CSF) production and absorption was disrupted, leading to ventricular dilation, the compression of neural structures, and subsequent functional impairments. The stages of hydrocephalus development and its etiology vary, resulting in different clinical symptoms [5,14,17]. The clinical symptoms observed in this study align with findings from previous research [2,5]; however, the incidence of strabismus in our data was relatively rare. Reketa (2011) noted that hydrocephalus caused by choroid plexus tumors is defined as communicating, while others are obstructive [18]. In this retrospective study, no cases of communicating hydrocephalus were found, which may be due to its relative rarity. In obstructive hydrocephalus, the ventricles are the first to dilate, particularly in the lateral ventricular region, and this dilation may subsequently extend to the anterior and posterior horns, potentially affecting the olfactory bulbs and optic nerves [14]. In this study, disorientation (27.74%) was attributed to the dilation of the lateral ventricles compressing neural structures. As CSF flows, it accumulates in the fourth ventricle, potentially elevating the cerebellar peduncles. This accumulation ultimately affects the third ventricle, leading to reduced and flattened thalamic adhesion. Vestibular signs are associated with the dilation of the fourth ventricle [19], which accounts for the ataxia (14.60%) and head tilt (9.49%) cases in this study, indicating significant clinical relevance. Abnormalities at the cranial–cervical junction are also associated with the fourth ventricle. Additionally, severe dilation of the lateral ventricles can compress the optic nerve pathways [3]. However, no significant damage to the optic nerves was observed in this study. Increased intracranial pressure may result in neural injury, manifesting as more pronounced clinical symptoms, but intracranial pressure was not investigated in this study. Therefore, the clinical manifestations of hydrocephalus are closely related to its etiology and the location of the lesion.

Hydrocephalus can be diagnosed using various imaging techniques, including ultrasound, X-ray, CT, and MRI [16]. Ultrasound is an effective method for examining open fontanelles in animals [19]. X-ray is another useful examination for assessing skull structure, but it can sometimes produce misleading results. CT is appropriate for initial screening [19,20], while MRI, with its superior soft tissue resolution, is essential for accurate diagnosis and treatment strategy formulation [19]. Therefore, our study focused on analyzing MRIs of hydrocephalus cases to minimize errors in hydrocephalus research and improve diagnostic accuracy. In human medicine, the Evans index is a key indicator in diagnosing hydrocephalus, particularly when its value exceeds 0.3 [15,21], as it accurately reflects the enlargement of the ventricular system. Additionally, enhanced indicators such as the Z-EI, VBA, and the angle of the corpus callosum offer a more comprehensive perspective for monitoring changes in the lateral ventricular system [21,22]. However, these indicators primarily target frontal horn expansion and require specific positioning for accurate measurement. Furthermore, cases involving the isolated expansion of the fourth ventricle are often at risk of being misdiagnosed [23]. In veterinary clinical practice, these indicators are challenging to implement, and isolated expansion of the fourth ventricle is common in animal patients. Therefore, our study underscored the necessity of developing and applying diagnostic tools and indicators tailored to the unique characteristics of animals in the veterinary field to enhance the accuracy and comprehensiveness of hydrocephalus diagnosis.

This study quantitatively analyzed the lateral ventricles and measured the middle region of the left and right lateral ventricles (thalamic adhesion). Although this method may reduce sensitivity, it enhances specificity [22,24]. Using the Mimics software 21.0, we accurately segmented the images of the lateral ventricles and proposed a novel perspective: irrespective of factors such as breed, weight, and age, the height and area-to-volume ratio at the thalamic adhesion level exhibit a significant relationship, which can indirectly indicate the degree of lateral ventricular expansion [25,26]. This finding offers a new approach to the quantitative assessment of hydrocephalus. Due to the absence of standardized protocols for animal MRI scans, this study aligned the coronal plane parallel to the skull base and positioned the sagittal plane perpendicularly to measure the height and volume relationship of the ventricles at the mid-position. Out of 137 cases of hydrocephalus, no significant differences were observed in area or volume compared with normal animals, making it difficult to distinguish hydrocephalus based on these parameters (Table 3 and Table 4). However, a significant difference in height was noted (Table 2). The simple measurement of height alone did not sufficiently capture the differences; therefore, the maximum percentage of ventricle height to brain height was chosen as the primary indicator [13]. In line with previous studies, the ratio of ventricular height to brain height was classified as normal size (0–14% Vh/Bh), moderately enlarged (15% to 25% Vh/Bh), or severely enlarged (>25% Vh/Bh) [11,27]. In this study, the range of maximum height percentages for hydrocephalus (15.14% to 76.44%) was significantly different from that of the normal group (6.5% to 13.18%), which is consistent with prior research. To establish a more accurate diagnostic score, a large-scale investigation of the height percentages of hydrocephalus in animals that closely resemble normal specimens is necessary. This will facilitate the construction of a receiver operating characteristic (ROC) curve to develop diagnostic standards for animals.

Based on the study by Esteve-Ratsch et al. (2001), the asymmetry of the left versus right ventricles was classified into three categories: slight (1:1.1 to 1.5), moderate (1.6 to 2:1), and extensive (>2:1) asymmetry [12]. In this study, extensive asymmetry (>2:1) was noted in 16 of the 137 cases. This finding indicates asymmetry in hydrocephalic cases, likely related to the site of blockage, the location of local lesions, and the developmental characteristics of the animals. In this study, the hydrocephalus group exhibited a significant difference in height compared with the normal group; however, the differences in area and volume were not pronounced, which is consistent with the findings of Woo et al. (2010). Compared with Woo’s equation [13], the data from this study exhibit a greater skew toward the *X*-axis, and the sample size is larger than in previous studies, resulting in enhanced specificity. A significant difference can be observed at a height ratio of 15% between normal and hydrocephalic animals.

The regression equation indicates that when the degree of ventricular expansion is less than 40%, the predicted values are highly correlated with the actual values. However, when the expansion degree exceeds 40%, the actual values may deviate from the predicted values, posing challenges in assessing the degree of hydrocephalus expansion and its impact on clinical symptoms. Furthermore, while ventricular expansion and hydrocephalus should be differentiated, evidence suggests that ventricular expansion may serve as a high-risk precursor to hydrocephalus. Simultaneously, a reduction in ventricular size can improve clinical symptoms, indicating a potential connection between the two conditions. Therefore, we recommend increasing the sample size in future studies to more accurately classify and understand these diseases. With advancements in MRI technology—such as the 3D-SPACE sequence—and the application of neuro-endoscopy, the misdiagnosis rate of hydrocephalus is expected to decrease [28,29].

This study employed three-dimensional (3D) modeling technology to conduct a quantitative analysis of 137 cases of canine hydrocephalus, examining the relationship between the height, area, and volume of the lateral ventricles at the site of thalamic adhesion. The derived regression equation offers a preliminary clinical screening tool for hydrocephalus diagnosis, particularly when combined with assessments of third and fourth ventricular enlargement and extra-ventricular conditions. The Spearman’s rank correlation coefficient found no significant correlation between clinical symptoms and height index (not present). While all data were clinically sourced, potential limitations include imbalances in breed, age, and gender distribution, as well as morphological variations in canine skull anatomy, which may influence the model’s generalizability. Furthermore, the study’s reliability is constrained by the hydrocephalus group’s sample size and the limited control cohort, inherent to clinical data collection challenges. Future applications of this model should account for these variables and integrate case-specific clinical contexts to optimize diagnostic accuracy.

## 5. Conclusions

We determined that the maximum percentage of ventricular height to brain height (H-max%) at the thalamic adhesion level can serve as an important indicator of the volume and area associated with hydrocephalus.

## Figures and Tables

**Table 1:** Retrospective analysis.

	Normal (n = 17)	Hydrocephalus (n = 137)	*p*-Value *
Year	6.24 ± 4.41	5.85 ± 4.77	*0.751340*
Body Weight	7.71 ± 2.82	6.13 ± 6.49	*0.325334*
Sex	Female: 8 Male: 9	Female: 73 Male: 64	
Clinical Symptoms		Disorientation: 27.74% (Dogs: 38) Ataxia: 14.60% (Dogs: 20) Head tilt: 9.49% (Dogs: 13)	

*: *p* < 0.05.

**Table 2:** Heights of left and right ventricles and brain.

	Normal (n = 17)	Hydrocephalus (n = 137)	*p*-Value *
VHl (mm)	3.17 ± 0.96	8.99 ± 5.42	*0.000020*
VHr (mm)	3.21 ± 0.92	8.59 ± 5.08	*0.000026*
BH (mm)	36.35 ± 5.20	31.81 ± 12.48	*0.140816*

Note: VHl: left ventricular height; VHr: right ventricular height; BH: brain height. *: *p* < 0.05.

**Table 3:** Areas of left and right ventricles and brain.

	Normal (n = 17)	Hydrocephalus (n = 137)	*p*-Value *
VAl (mm^2^)	18.13 ± 8.31	123.10 ± 215.00	0.046458
VAr (mm^2^)	17.52 ± 8.59	119.80 ± 211.70	0.048792
BA (mm^2^)	1279.00 ± 482.00	1747.00 ± 985.30	0.055857

Note: VAl: left ventricular area; VAr: right ventricular area; BA: brain area. *: *p* < 0.05.

**Table 4:** Volumes of left and right ventricles and brain.

	Normal (n = 17)	Hydrocephalus (n = 137)	*p*-Value *
VVl (mm^3^)	506.50 ± 386.20	2755.00 ± 6247.00	*0.141055*
VVr (mm^3^)	493.10 ± 370.30	2646.00 ± 6194.00	*0.155121*
BV (mm^3^)	61,134.00 ± 21,383.00	50,906.00 ± 31,346.00	*0.193463*

Note: VVl: left ventricular volume; VVr: right ventricular volume; BV: brain volume. *: *p* < 0.05.

**Table 5:** Spearman correlation.

Correlation			H-Max%	A-Max%	V-Max%
Spearman	H-max%	Correlation coefficient	*1*	*0.894* **	*0.792* **
		Sig. (two-tailed)	*0.000*	*0.000*	*0.000*
		N	137	137	137
	A-max%	Correlation coefficient	*0.883* **	*1*	*0.777* **
		Sig. (two-tailed)	*0.000*	*0.000*	*0.000*
		N	137	137	137
	V-max%	Correlation coefficient	*0.792* **	*0.796* **	*1*
		Sig. (two-tailed)	*0.000*	*0.000*	*0.000*
		N	137	137	137

** Correlation is significant at the 0.01 level (two-tailed).

## Data Availability

The data presented in this study are available upon request from the corresponding author.
